# Efficacy and Safety of Botulinum Toxin in the Management of Temporomandibular Symptoms Associated with Sleep Bruxism: A Systematic Review

**DOI:** 10.3390/dj12060156

**Published:** 2024-05-23

**Authors:** Roxana Buzatu, Magda Mihaela Luca, Luca Castiglione, Cosmin Sinescu

**Affiliations:** 1 Department of Dental Aesthetics, Faculty of Dental Medicine, “Victor Babes” University of Medicine and Pharmacy Timisoara, Revolutiei Boulevard 9, 300041 Timisoara, Romania; roxana.buzatu@umft.ro; 2 Department of Pediatric Dentistry, Faculty of Dental Medicine, “Victor Babes” University of Medicine and Pharmacy Timisoara, Eftimie Murgu Square 2, 300041 Timisoara, Romania; 3 Doctoral School, Faculty of General Medicine, “Victor Babes” University of Medicine and Pharmacy Timisoara, Eftimie Murgu Square 2, 300041 Timisoara, Romania; castiglione.luca@umft.ro; 4 Department of Prostheses Technology and Dental Materials, Faculty of Dental Medicine, “Victor Babes” University of Medicine and Pharmacy Timisoara, Eftimie Murgu Square 2, 300041 Timisoara, Romania; minosinescu@gmail.com

**Keywords:** botulinum toxin, Botox, temporomandibular joint disorders, bruxism, pain management, stomatology

## Abstract

Sleep bruxism, characterized by involuntary grinding or clenching of teeth during sleep, poses significant challenges in management due to its potential to induce temporomandibular joint disorders (TMDs) and other related symptoms. The use of Botulinum toxin Type A (BoNT-A), also known as Botox^®^, has been proposed as a therapeutic intervention. This systematic review aims to evaluate the efficacy and safety of BoNT-A in the management of sleep bruxism, focusing on pain reduction, improvement in jaw function, reduction in bruxism episodes, and the incidence of adverse effects. An exhaustive search was conducted across PubMed, Scopus, and Embase databases up to January 2024, adhering to the PRISMA guidelines. Nine randomized clinical trials (RCTs) involving 137 participants were analyzed for efficacy and safety outcomes. The studies demonstrated a significant reduction in mean pain scores (from 7.1 to 0.2 at 6 months and 1 year post-treatment in one study) and a notable decrease in the number of bruxism events (from 4.97/h to 1.70/h in the BoNT-A group in another study). Additionally, improvements were observed in jaw stiffness and total sleep time. Adverse effects varied but were generally mild and transient, including injection site pain in 20% of participants in one study and cosmetic changes in smile in 15.4% of patients in another. These findings suggest that BoNT-A injections may provide some benefits for treating nocturnal bruxism, potentially reducing TMD symptoms like pain and improving jaw function. However, these findings are preliminary due to variability in study designs and the absence of detailed statistical analysis.

## 1. Introduction

Temporomandibular disorders (TMDs) encompass a diverse group of conditions characterized by pain and dysfunction in the jaw joint and the muscles responsible for jaw movement. These disorders are a significant health concern due to their prevalence and impact on quality of life. TMDs are estimated to affect about 5% to 12% of the population, with a higher incidence among younger adults, particularly females [1].

Bruxism is clinically classified into two main types: sleep bruxism and awake bruxism. Sleep bruxism is considered a sleep-related movement disorder, where individuals involuntarily grind or clench their teeth during sleep. This type of bruxism is often associated with other sleep disorders, such as sleep apnea, and can significantly disrupt sleep quality [2]. Awake bruxism, on the other hand, involves the clenching or grinding of teeth while awake and is more commonly triggered by stress, anxiety, or concentration. Although the exact prevalence of bruxism is challenging to ascertain due to varying diagnostic criteria and methods of assessment, it is estimated that about 8% to 31% of the general population experiences bruxism to some degree. The condition seems to affect both genders equally, but certain age groups, particularly young adults, are more frequently diagnosed with bruxism [3].

The occurrence of bruxism can lead to a plethora of dental and systemic health issues, including the development of TMDs, excessive tooth wear, and even changes in facial aesthetics. Understanding the nature and impact of bruxism is crucial for developing effective management strategies for associated conditions like TMD. As such, the interrelationship between bruxism and TMD is an area of significant clinical interest, necessitating detailed exploration and targeted therapeutic approaches [4]. These include stress management, biofeedback, and relaxation techniques designed to reduce jaw tension, alongside the use of oral splints. Oral splints, often referred to as occlusal appliances or night guards, are custom-fitted devices worn to prevent tooth contact and mitigate the effects of clenching and grinding. However, the efficacy of these treatments is often variable and sometimes insufficient for providing long-term relief [5,6,7].

In recent years, Botulinum toxin Type A (BoNT-A) has been adopted as a novel approach for managing temporomandibular disorder (TMD) [8,9]. Known for its muscle-relaxant properties, BoNT-A targets key masticatory muscles: the masseter, which aids in chewing and clenching; the temporalis, involved in jaw closing; and the pterygoid muscles, which control jaw movements. This targeted approach helps reduce the severity of bruxism and alleviate TMD symptoms by blocking acetylcholine release at the neuromuscular junction, diminishing muscle contraction and spasticity [10,11,12,13,14].

The application of BoNT-A in this context is supported by a growing body of evidence from randomized controlled trials and observational studies that have reported on various outcomes, including reductions in pain levels, improvements in mouth opening range, and decreases in muscle tenderness [15,16]. However, the literature is not without its discrepancies, with studies varying in terms of methodology, injection protocols, and assessment criteria, thus necessitating a systematic review to consolidate the evidence. Moreover, the safety profile of BoNT-A when used for TMD and bruxism is of paramount concern. While generally considered safe, potential adverse effects related to the injection site or systemic spread of the toxin must be meticulously evaluated [17,18]. The balance between efficacy and safety is essential in determining the viability of BoNT-A as a standard treatment modality for these conditions.

Given the importance of treating sleep bruxism with its associated TMD symptoms that have a significant impact on oromandibular dental health, the current systematic review aims to evaluate the studies focusing on the efficacy and safety of botulinum toxin in the management of sleep bruxism. This is particularly important as clinicians seek to optimize treatment strategies, ensuring they are both evidence-based and aligned with patient-centric outcomes.

## 2. Materials and Methods

### 2.1. Protocol and Registration

For this systematic review, an extensive search strategy was executed across PubMed, Scopus, and Embase databases to encompass literature up to 12 January 2024, the initial search date. The search was aimed at identifying the latest studies on the application of Botulinum toxin Type A (BoNT-A), for the treatment of sleep bruxism. Making a distinction between awake and sleep bruxism in research studies is essential due to the differing etiologies, manifestations, levels of consciousness, and potential treatment strategies for these conditions, which may significantly impact the effectiveness of interventions.

The search was conducted by a team of three researchers. To ensure thoroughness and consistency, each article identified was independently reviewed by two team members. In cases of disagreement regarding the eligibility of a study, the issue was resolved through discussion among the team members. If consensus could not be reached, a third senior researcher was consulted to make the final decision.

The PICO framework guided the review, as presented in Table 1.

Following the PRISMA guidelines [19], this protocol was developed with a focus on transparency, structure, and reproducibility. The review has been registered with the Open Science Framework with the registration number found at https://osf.io/mx3jb, emphasizing our commitment to a rigorous systematic review process. The detailed and comprehensive search strategy aims to ensure that all pertinent studies are identified, contributing significantly to the understanding and evidence base surrounding the use of BoNT-A in treating sleep bruxism.

### 2.2. Eligibility Criteria and Definitions

The eligibility criteria for this systematic review were developed to identify studies that examined the efficacy and safety of Botulinum toxin Type A (BoNT-A), also known as Botox, in the treatment of sleep bruxism and its associated TMD symptoms. The inclusion criteria for this review were delineated as follows: Firstly, the study population had to consist of individuals diagnosed with sleep bruxism, without restrictions on age or sex, to encompass a broad demographic affected by these conditions. Secondly, the research had to explicitly investigate the use of BoNT-A as an intervention for managing symptoms of TMD and sleep bruxism. This included studies evaluating the effects of BoNT-A on pain reduction, improvement in jaw function and jaw motor events, masseteric and myofascial pain, headaches, chronic pain, and any TMD and adverse effects related to treatment. Thirdly, only randomized controlled trials were considered for inclusion. Fourthly, only studies utilizing validated assessment tools or well-defined parameters for measuring treatment outcomes were included. Finally, the review focused on peer-reviewed articles published in English.

Conversely, studies were excluded if they met any of the following criteria: non-human studies, such as in vitro or animal research, to concentrate exclusively on outcomes relevant to human patients; and studies that did not specifically differentiate between awake and sleep bruxism or failed to adequately report on the outcomes of interest, such as pain relief, functional improvement, or adverse effects. Additionally, research lacking specific, quantifiable outcomes related to the efficacy and safety of BoNT-A treatment or missing sufficient detail for evaluation was excluded. Moreover, observational studies, cohort studies, case–control studies, and quasi-experimental studies were excluded from the final analysis. Lastly, non-peer-reviewed articles, conference proceedings, theses, dissertations, general reviews, commentaries, and editorials were also excluded to ensure the review was based on scientifically valid and peer-evaluated sources.

### 2.3. Data Collection Process

**Figure 1:**
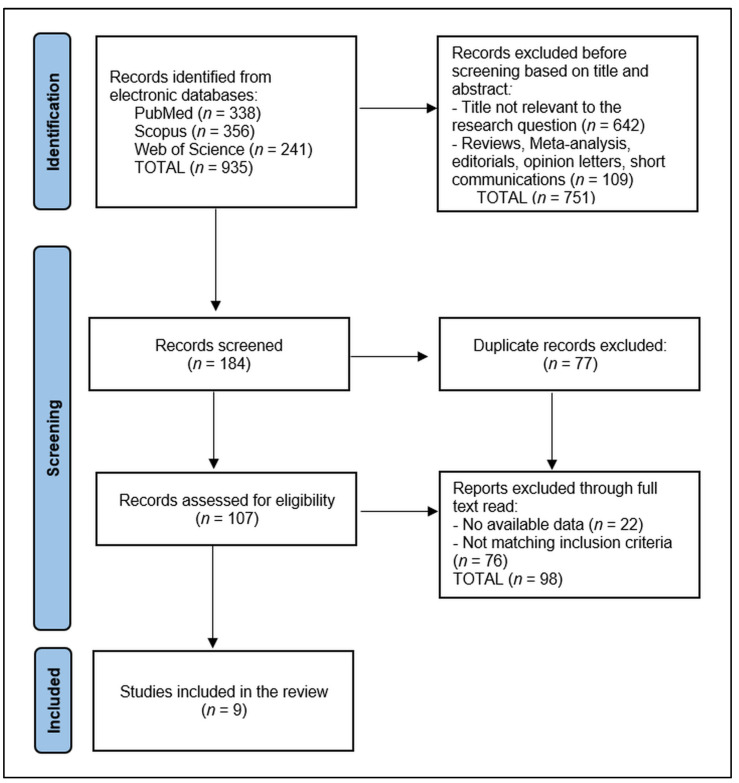
PRISMA flow diagram.

The data collection process for this systematic review commenced with the removal of 77 duplicate entries, followed by a rigorous screening of 184 abstracts by two independent reviewers to assess each study’s relevance based on predefined inclusion and exclusion criteria. Of 107 records assessed for eligibility, 22 were excluded for having no available data (n = 76) or for not matching the inclusion criteria regarding the permanent dentition, age of the patients, and serum vitamin D assessment. Discrepancies between reviewers were resolved through discussion or, if necessary, consultation with a third reviewer to achieve consensus. The initial database search yielded 935 articles, from which 9 relevant studies were identified for inclusion in the final study, as presented in Figure 1.

### 2.4. Risk of Bias and Quality Assessment

For the systematic assessment of study quality and determination of risk of bias within the included studies, our review employed a dual approach, integrating both qualitative and quantitative evaluation methods. Initially, the quality of observational studies was evaluated by the researchers R.B. and L.C. using the Newcastle–Ottawa Scale [20]. Discrepancies in quality assessment scores were resolved through discussion or, if necessary, consultation with a third researcher.

## 3. Results

### 3.1. Study Characteristics

The systematic review included a total of nine studies [21,22,23,24,25,26,27,28,29], as summarized in Table 2. These studies were conducted across various countries, including Saudi Arabia [21,28], Italy [22], the United States [23,24], South Korea [25,27], Syria [26], and Australia [29], over a period spanning from 2010 to 2022. All selected studies utilized a randomized clinical trial design, focusing on the effects of Botulinum toxin Type A in the treatment of sleep-bruxism-associated TMD symptoms. The assessment of study quality revealed a mixture of medium- and high-quality ratings, with most studies being classified as medium quality [21,24,25,26,27,28] and three studies [22,23,29] receiving a high-quality rating.

### 3.2. Population Characteristics

The systematic review analyzed population characteristics across nine studies, as shown in Table 3, collectively involving a total of 137 participants in the BTX-A intervention groups. The participant age in these studies varied broadly, with a mean age range from 24.8 in the Lee et al. study [23] to 48.6 years in the study by Ondo et al. [24]. Gender distribution varied across the studies, with a notable predominance of female participants in several studies, such as the one by Al-Wayli et al. [21] with 100% female participants, and Ondo et al. [24] with 76.9% female participants.

Study groups within these trials were categorized based on the type of intervention received, including conventional treatments, placebo injections, and various botulinum toxin injection protocols targeting different muscle groups involved in bruxism. For instance, Shim et al. [25] divided their sample into two groups, with one receiving injections solely in the masseter muscle and the other receiving injections in both the masseter and temporalis muscles, allowing for a comparative analysis of the outcomes based on the injection sites.

### 3.3. Clinical Trial Assessment

Dose variations were significant, with Al-Wayli et al. [21] employing 20 units of BTX-A per side, whereas Ondo et al. [24] used a substantial 200 units distributed between the masseter and anterior temporalis muscles. Three other studies [25,27,28] administered 25 units of BTX-A across individual muscle injection sites.

Follow-up periods varied extensively among the studies, from as short as one week in Guarda-Nardini et al. [22] to as long as one year in Al-Wayli et al. [21], providing a comprehensive view of BTX-A’s efficacy and safety over varying durations. The injection sites predominantly targeted the masseter muscle, with several studies extending treatment to the anterior temporalis muscles [22,24], and Cruse et al. [29] further including the medial pterygoid muscles.

Adverse effects were reported with varying frequency across the studies. Notably, Ondo et al. [24] observed cosmetic changes in 15.4% of patients, highlighting the potential for visible side effects with higher BTX-A dosages. Shim et al. [25] reported discomfort and masticatory difficulties in a significant proportion of their patients, underlining the importance of considering patient comfort and functional outcomes in treatment plans. Shehri et al. [26] noted injection site pain in 20% of participants, a reminder of the need for careful technique and possibly patient counseling on what to expect post-treatment. Cruse et al. [29] found that 22.7% of patients experienced mild and transient side effects, suggesting that while BTX-A treatment is generally well tolerated, patient monitoring and follow-up are essential for managing and mitigating adverse effects. Five other studies reported no adverse effects [21,22,23,27,28], as presented in Table 4.

### 3.4. Assessment of Outcomes

Al-Wayli et al. [21] demonstrated a remarkable reduction in mean pain scores, from 7.1 pre-treatment to 0.2 at 6 months and 1 year post-treatment in the BTX-A group. Guarda-Nardini et al. [22] observed a modest improvement in pain at rest, with scores decreasing from 5.00 at baseline to 3.60 in the BTX-A group, compared to a slight increase from 3.90 to 4.10 in the placebo group at six months. Lee et al. [23] did not report pain results but noted a decrease in bruxism symptoms at 12 weeks, with a significant reduction in the number of bruxism events from 4.97/h to 1.70/h in the BTX-A group, contrasting with an increase in the placebo group from 4.24/h to 4.83/h, indicating BTX-A’s potential to specifically target and reduce bruxism activity.

Ondo et al. [24] reported a Visual Analog Scale (VAS) pain score at 4 weeks post-treatment that remained high at 65.0 in the BTX-A group compared to 44.2 in the placebo group. However, they observed an improvement in total sleep time in the BTX-A group, suggesting secondary benefits of treatment despite the pain scores. Shim et al. [25] found a reduction in morning jaw stiffness of 47.50% in the group receiving masseter muscle injections only, and 57.50% in the group treated in both masseter and temporalis muscles. Moreover, 45.0% of patients reported a reduction in tooth grinding at 4 weeks post-injection, underlining BTX-A’s effectiveness in alleviating symptomatic stiffness and bruxism activity.

Shehri et al. [26] noted significant pain reduction, with VAS pain scores decreasing from 8.62 to 6.07 in the BTX-A group at 6 months post-treatment versus an increase in the placebo group from 8.42 to 8.62, highlighting the analgesic effect of BTX-A in sleep bruxism. Alwayli et al. [28] and Cruse et al. [29] further corroborated the pain-reducing impact of BTX-A, with significant decreases in mean pain scores observed over their respective follow-up periods, emphasizing BTX-A’s role in managing pain associated with sleep bruxism (Table 5 and Supplementary File).

## 4. Discussion

### 4.1. Summary of Evidence

This systematic review revealed Botulinum toxin Type A as a notably effective treatment for reducing pain in TMD associated with sleep bruxism. The significant reduction in mean pain scores, as observed in several studies, underscores BTX-A’s efficacy in alleviating chronic myofascial and temporomandibular joint pain. The consistency of pain reduction across various dosages and injection sites illustrates BTX-A’s potential as a versatile treatment option. However, the absence of significant improvement in control groups across these studies highlights the specificity of BTX-A’s therapeutic effects compared to other interventions.

While BTX-A demonstrated a profound impact on pain management, its effect on other clinical outcomes related to bruxism, such as the reduction in the frequency of bruxism events and muscle activity, presents an area ripe for further exploration. For instance, the decrease in bruxism symptoms and the number of bruxism events in some studies suggest a direct influence of BTX-A on the underlying mechanisms of bruxism. However, the lack of significant improvements in mouth opening and maximum occlusal force in certain studies points to the complex nature of bruxism as a condition that may require a multifaceted treatment approach beyond muscle relaxation.

The varied dosages and follow-up periods reported across studies raise important considerations regarding the optimal treatment regimen for BTX-A in managing sleep bruxism and TMD. The wide range of BTX-A doses, from as low as 10 units to as high as 200 units, alongside diverse injection sites, reflects the tailored approach to treatment but also indicates the need for standardized guidelines to maximize efficacy and minimize adverse effects.

Adverse effects associated with BTX-A were generally minimal and transient, supporting its safety profile. However, the presence of discomfort, masticatory difficulties, and cosmetic changes in a minority of patients underscores the necessity of a cautious approach to treatment, emphasizing patient counseling and the careful consideration of dosage and injection sites. The transient nature of some treatment effects, with symptoms gradually resurfacing, suggests that BTX-A treatment for sleep bruxism and TMD may require ongoing management rather than a one-time intervention.

Several studies on the application of BTX-A in the treatment of temporomandibular myofascial pain and sleep bruxism were excluded from this systematic review due to their design not meeting the criteria for clinical trials. Despite their exclusion, their findings offer valuable insights into BTX-A’s therapeutic potential. For instance, Hosgor et al. [30] conducted a clinical record review of 44 patients, demonstrating significant improvements in the range of jaw motion and reductions in pain levels, as measured by the VAS, over a six-month follow-up period. These improvements were observed in unassisted maximum mouth opening, protrusion, and right and left laterotrusion, suggesting BTX-A’s efficacy in enhancing functional outcomes alongside pain management. Similarly, Asutay et al.’s [31] retrospective study included 25 female patients, focusing on the efficacy of BTX-A for sleep bruxism in those unresponsive to conservative treatments. This study also reported significant pain relief following BTX-A injections in the masseters, with minimal adverse events noted.

Similarly, two systematic reviews that were not included in our study shed light on the complexity of using BTX-A for TMDs. Delcanho et al. [32] analyzed 24 randomized clinical trials, highlighting that BTX-A injections showed superiority over placebo in reducing TMD pain levels and improving maximum mouth opening in a total of 411 patients across varied interventions. However, Saini et al.’s review [33] of 14 RCTs with 395 patients revealed that the effectiveness of BTX-A in pain reduction was not significantly better than placebo, with mean differences in pain scores at −1.71 (95% CI, −2.87 to −0.5) at one month, −1.53 (95% CI, −2.80 to −0.27) at three months, and −1.33 (95% CI, −2.74 to 0.77) at six months. Both reviews call attention to the nuanced efficacy of BTX-A in TMD management and the critical need for more rigorous trials to definitively establish treatment protocols and outcomes.

Thambar et al.’s [34] investigation into the efficacy of botulinum toxin for TMD and masticatory myofascial pain, based on a review of seven studies, revealed mixed results: three studies showed significant pain reduction between BTX and placebo groups, while others reported no significant difference or equal pain reduction with alternative treatments. Similarly, Di Francesco et al.’s systematic review of 11 randomized controlled trials highlighted BoNT-A as a potential option for patients unresponsive to conservative TMD treatments, recommending low doses for managing persistent orofacial pain [35]. Both reviews underscore the complex efficacy profile of BTX in TMD management and emphasize the need for more rigorous, large-scale studies to clarify its therapeutic benefits and optimal application protocols, reflecting the broader scientific community’s call for nuanced, evidence-based approaches to utilizing BTX in TMD treatment.

Kaya’s study on 40 bruxism patients compared occlusal splinting with botulinum toxin injections, revealing both treatments effectively reduced pain without a significant difference in efficacy [36]. Notably, BTX-A led to a temporary reduction in maximum bite force, which decreased in the 2nd and 6th weeks but increased in the 3rd and 6th months. Similarly, Kef et al. [37] observed in their study of 37 patients with secondary otalgia from bruxism that BTX-A injections significantly alleviated complaints within two weeks. Furthermore, 24.3% of patients presenting with facial asymmetry due to masseter hypertrophy experienced a decrease in asymmetry, showing noticeable improvement by the 4th month. These findings quantitatively highlight BTX-A’s role not only in pain management but also in improving functional and aesthetic outcomes in bruxism-related conditions.

Similarly, Chen et al.’s systematic review [38] on the efficacy of botulinum toxin A for bruxism included a meta-analysis from ten studies, demonstrating a significant reduction in maximal biting force and pain severity with BTX-A injections compared to oral splints and saline injections. This effect was most pronounced within the first month, continuing to outperform other methods at 3 months, and showed that higher doses of BTX correlated with greater pain improvement. Conversely, Sendra et al.’s [39] systematic review, examining clinical outcomes of BTX injections for primary bruxism, analyzed six randomized clinical trials and four case series. Despite the heterogeneity that precluded a meta-analysis, all selected studies affirmed the efficacy and safety of BTX injections in reducing symptoms of primary bruxism. Both reviews advocate for BTX as a valuable treatment option for bruxism, particularly for those unresponsive to traditional therapies, although they also emphasize the need for further randomized trials to refine treatment protocols.

In examining the efficacy of Botulinum toxin Type A (BTX-A) in managing sleep bruxism, our findings resonate with several aspects of previous studies yet provide unique insights due to our specific focus. For instance, the meta-analysis by Yun Chen et al. [38] underscores the general effectiveness of BTX-A across various types of bruxism, aligning with our observation of pain reduction and improved jaw function in sleep cases. Similarly, the study by Saini et al. [33] concludes that BTX-A is effective for TMD, which is often a consequence of sleep bruxism, suggesting overlapping benefits. However, our analysis delves deeper into the nocturnal-specific manifestations and treatments, an area less explored by these broad reviews. Furthermore, the *British Journal of Oral and Maxillofacial Surgery* (2022) highlights the reduction in bruxism events, a point our review elaborates on by distinguishing the effects seen specifically at night [40]. These comparisons elucidate the broader applicability of BTX-A, while our focused approach on nocturnal symptoms helps refine the understanding of treatment timings and potential for targeted therapeutic interventions. This detailed exploration within our discussion helps bridge the gap between generalized bruxism treatments and the nuanced needs of sleep bruxism sufferers, paving the way for more specialized management strategies.

The clinical utility of these findings in dentistry is substantial, offering a promising intervention strategy to mitigate the adverse effects of sleep bruxism. By demonstrating the efficacy and safety of Botulinum toxin Type A (BoNT-A) in reducing pain, improving jaw function, and decreasing bruxism episodes, this systematic review provides dentists with an evidence-based treatment option. Utilizing BoNT-A as part of a comprehensive management plan can help avoid the long-term complications associated with untreated bruxism, such as tooth wear, increased risk of TMD, and myofascial pain. Incorporating BoNT-A injections into clinical practice not only addresses the immediate symptoms of bruxism but also potentially prevents the progression of associated dental complications, enhancing overall patient quality of life and dental health.

### 4.2. Limitations

This systematic review’s insights are constrained by several critical limitations. The heterogeneity across studies regarding BTX-A dosages, injection sites, follow-up durations, and participant demographics, notably the predominance of female participants, introduces challenges in drawing uniform conclusions and formulating standardized treatment protocols. Moreover, the small sample sizes limit the statistical power and generalizability of the findings. A meta-analysis was not conducted due to the substantial variability in outcome measures, treatment protocols, and reporting styles across the included studies. This heterogeneity, evidenced by differences in pain measurement scales, variations in follow-up periods, and inconsistent reporting of secondary outcomes, made it challenging to aggregate data quantitatively in a meaningful way. These limitations highlight the need for more extensive, diverse, and methodologically uniform studies to fully understand BTX-A’s therapeutic potential and establish evidence-based guidelines for its use in treating TMD and sleep bruxism.

## 5. Conclusions

This systematic review indicates that Botulinum toxin Type A (BTX-A) injections may provide some benefits for treating nocturnal bruxism, potentially reducing TMD symptoms like pain and improving jaw function. However, these findings are preliminary due to variability in study designs and the absence of detailed statistical analysis. The evidence supports the cautious integration of BTX-A into treatment plans, but more rigorous research is needed to confirm its efficacy and safety. Future studies should focus on larger cohorts and standardized methodologies to provide a clearer understanding of BTX-A’s role in managing bruxism.

## Figures and Tables

**Table 1:** PICO framework.

Study and Author	Country
Population	Adults diagnosed with temporomandibular disorder (TMD) and bruxism.
Intervention	Use of Botulinum toxin Type A (BoNT-A) for managing symptoms.
Comparison	Comparisons include placebo treatments, traditional therapies such as oral splints, or no treatment.
Outcome	Reduction in pain levels, improvements in jaw function, decrease in muscle tenderness, and assessment of adverse effects.

**Table 2:** Study characteristics.

Study and Author	Country	Study Year	Study Design	Study Quality
1 [21] Al-Wayli et al.	Saudi Arabia	2017	Randomized clinical trial	Medium
2 [22] Guarda-Nardini et al.	Italy	2014	Randomized clinical trial	High
3 [23] Lee et al.	United States	2010	Randomized clinical trial	High
4 [24] Ondo al.	United States	2018	Randomized clinical trial	Medium
5 [25] Shim et al.	South Korea	2014	Randomized clinical trial	Medium
6 [26] Shehri et al.	Syria	2022	Randomized clinical trial	Medium
7 [27] Shim et al.	South Korea	2020	Randomized clinical trial	Medium
8 [28] Alwayli et al.	Saudi Arabia	2021	Randomized clinical trial	Medium
9 [29] Cruse et al.	Australia	2022	Randomized clinical trial	High

**Table 3:** Population characteristics.

Study Number	Sample Size (Intervention Group)	Age/Age Range	Gender Distribution	Control Group
1 [21] Al-Wayli et al.	25	Mean: 45.5 ± 10.8 years	Female: 100%	Patients treated with saline placebo injection (n = 25)
2 [22] Guarda-Nardini et al.	10	Mean: 38 ± 10.8 (25–45) years	Female: 50% Male: 50%	Patients treated with saline placebo injection (n = 10).
3 [23] Lee et al.	6	Mean: 25.0 ± 2.35 years for men and 24.8 ± 0.83 years for women	Female: 58.3% Male: 41.7%	Patients treated with saline placebo injection (n = 6).
4 [24] Ondo et al.	13	Mean: 48.6 ± 13.6 (20–30) years	Female: 76.9% Male: 23.1%	Patients treated with saline placebo injection (n = 10).
5 [25] Shim et al.	20	Mean: 25.8 ± 5.1 (20–38) years	Female: 50% Male: 50%	No control group
6 [26] Shehri et al.	20	Mean: 29.81 ± 7.12 (18–40) years	Female: 65.0% Male: 35.0%	Patients treated with sham intervention (n = 11)
7 [27] Shim et al.	13	Mean: 32.46 ± 9.94 (20–56) years	Female: 45.0% Male: 55.0%	Patients treated with saline placebo injection (n = 10).
8 [28] Alwayli et al.	20	Mean: 39.9 (21–52) years	Female: 80.0% Male: 20.0%	Patients treated with saline placebo injection (n = 200).
9 [29] Cruse et al.	22	Mean: 42.1 (22–68) years	Female: 63.6% Male: 36.4%	No control group

**Table 4:** Clinical trial assessment.

Study Number	Dose	Follow-Up	Injection Site	Adverse Effects
1 [21] Al-Wayli et al.	20 units of BTX-A per side	3 weeks, 2 months, 6 months, and 1 year	Masseter muscle bilaterally at 3 points	0%
2 [22] Guarda-Nardini et al.	30 units of BTX-A in the masseter muscle and three injections within the anterior temporalis muscles (20 units each), totaling 100 units.	1 week, 1 month, and 6 months	Masseter muscles and anterior temporalis muscles bilaterally	0%
3 [23] Lee et al.	80 units of BTX-A	4 weeks, 8 weeks, and 12 weeks	Masseter muscle bilaterally at 3 points	0%
4 [24] Ondo et al.	200 units of BTX-A (60 into each masseter and 40 into each temporalis)	4 to 8 weeks	Masseter muscles and anterior temporalis muscles bilaterally	2 patients (15.4%) experienced cosmetic changes in their smile
5 [25] Shim et al.	25 units of BTX-A per muscle	4 weeks	Group A: Masseter muscles only. Group B: Both masseter and temporalis muscles.	14 patients (70.0%) with discomfort associated with a decrease in the sensation of masticatory force, and 3 patients (15.0%) with masticatory difficulties.
6 [26] Shehri et al.	10 units of BTX-A per side	2 weeks, 1 month, 3 months, and 6 months	Masseter muscle bilaterally	4 patients (20%) experienced pain at injection site one week after injection.
7 [27] Shim et al.	25 units of BTX-A per masseter muscle	4 weeks and 12 weeks	Masseter muscle bilaterally	0%
8 [28] Alwayli et al.	25 units of BTX-A per masseter muscle	2, 4, 8, 12, 16, 18, and 24 weeks	Masseter muscle bilaterally	0%
9 [29] Cruse et al.	Group A: Bilateral masseter (60 units (U); Group B: Bilateral masseter and temporalis (90 U); Group C: Bilateral masseter, temporalis, and medial pterygoid muscles (120 U)	4 weeks and 12 weeks	Bilateral masseter; Bilateral masseter and temporalis; Bilateral masseter, temporalis, and medial pterygoid muscles.	5 patients (22.7%) experienced mild and transient side effects.

BTX-A—Botulinum toxin type A.

**Table 5:** Assessment of outcomes.

Study Number	Pain Results	Other Outcomes	Interpretation
1 [21] Al-Wayli et al.	Significant reduction in mean pain score from 7.1 ± 0.72 (pre-treatment) to 0.2 ± 0.51 (at 6 months and 1-year post-treatment) in the botulinum toxin injection group. No significant improvement in the control group.	NR	BTX-A was significantly more effective and safer as treatment for sleep bruxism associated with chronic myofascial and temporomandibular joint pain.
2 [22] Guarda-Nardini et al.	Improvements in pain at rest from 5.00 ± 3.62 at baseline to 3.60 ± 2.88 for BTX vs. from 3.90 ± 2.92 at baseline to 4.10 ± 2.85 for placebo at six months. Reduction in pain during chewing from 6.20 ± 2.78 at baseline to 3.60 ± 2.37 for the BTX group vs. 4.10 ± 2.92 at baseline to 4.70 ± 2.79 for placebo at six months.	No significant differences in the range of mouth opening, assisted and non-assisted	BTX-A did not significantly improve mouth opening, maximum occlusal force, or decrease bruxism events compared to placebo and other treatments.
3 [23] Lee et al.	NR	Bruxism symptoms at 12 weeks decreased from 1.75 ± 0.91 to 0.61 ± 0.64, compared with placebo (from 1.89 ± 0.71 to 1.39 ± 1.00)	The number of bruxism events at 12 weeks in the BTX-A group decreased from 4.97 ± 2.33/h to 1.70 ± 0.91/h, while in the placebo group increased from 4.24 ± 2.25/h to 4.83 ± 2.62/h
4 [24] Ondo et al.	VAS pain score at 4 weeks: 65.0 ± 19.6 (BTX-A) vs. 44.2 ± 14.3 (placebo)	Total sleep time tended to improve more in the BoNT group (increased 34.3 ± 58.6 min vs. decreased 11.7 ± 53 min in the placebo group.	The number of bruxism events in the BTX-A group decreased from 9.18 ± 8.48/h to 6.95 ± 7.04/h, while in the placebo group, events increased from 4.63 ± 3.45/h to 10.65 ± 9.57/h
5 [25] Shim et al.	Reduction in morning jaw stiffness after 47.50 ± 15.86% in Group A (masseter muscle injection only) and 57.50 ± 30.30% in Group B (masseter and temporal muscle)	Group A (masseter muscle injection only) achieved significantly shorter REM sleep of 14.38 ± 4.38% of total sleep, compared with 19.72 ± 9.72% in Group B (masseter and temporal muscle)	At 4 weeks after injection, 9 (45.0%) patients self-reported a reduction in tooth grinding. A single BTX-A injection effectively manages sleep bruxism for over a month by decreasing the intensity, not the occurrence, of jaw-closing muscle contractions.
6 [26] Shehri et al.	VAS pain score at 6 months: decrease from 8.62 ± 1.35 to 6.07 ± 1.05 (BTX-A) vs. 8.42 ± 0.67 to 8.62 ± 0.51 (placebo)	The mean EMG records of muscular activity were statistically smaller in the BTX-A group compared to the placebo. The mean time at which the loss of effectiveness started was 3.5 months.	Injecting 10 units of BTX-A into the masseter muscle effectively reduced muscle activity and pain associated with sleep bruxism for approximately three months before symptoms gradually relapsed
7 [27] Shim et al.	NR	The mean EMG records of muscular activity were statistically smaller in the BTX-A group (from 89.23 µVto 36.69 µV, compared to the placebo group (from 118.0 µVto 92.0 µV)	The study concluded that a single BoNT-A injection did not reduce the genesis of sleep bruxism but significantly reduced the intensity of masseter muscle contractions for up to 12 weeks.
8 [28] Alwayli et al.	The mean pain score at 8 weeks postoperatively in group A was 2.2 ± 0.59 and in group B was 5.2 ± 0.38. The initial mean VPS score was 5.75, which decreased to 0.44 after two weeks and then gradually increased to 2.00 at 24 weeks.	NR	BTX-A injections significantly reduced pain associated with sleep bruxism over the study period. The effects were most pronounced in the first 16 weeks, with a mild increase in pain scores noted up to the 24-week follow-up.
9 [29] Cruse et al.	VAS pain score at 12 weeks: decrease from 56.9 ± 26.7 to 44.6 ± 27.1 (BTX-A) vs. 56.9 ± 26.7 to 53.9 ± 29.9 (placebo)	The Bruxism Index was significantly lower at 4 weeks after active treatment when compared with placebo (mean = −1.66); however, this was not sustained at 12 weeks.	A greater benefit may be achieved by administering BTX-A into more muscles and at higher total doses, especially among those with higher baseline Bruxism Index.

NR—not reported; VAS—Visual Analog Scale.

## Data Availability

Not applicable.

## Supplementary Materials

The following supporting information can be downloaded at: https://www.mdpi.com/article/10.3390/dj12060156/s1.
