# Supplementary material for: Efficacy and Safety of Botulinum Toxin in the Management of Temporomandibular Symptoms Associated with Sleep Bruxism: A Systematic Review
Source: Dent J (Basel). 2024 May 23;12(6):156. doi: 10.3390/dj12060156 (PMC11203296; doi:10.3390/dj12060156)
Supplement: Supplementary file 1 [file dentistry-12-00156-s001.zip › dentistry-2965373-supplementary.pdf]

An expanded set of keywords and phrases was developed to thoroughly explore the intersection of BoNT-A treatment and TMD-related bruxism. These included: "Botulinum Toxin Type A," "Botox®," "Temporomandibular Joint Disorders," "Bruxism," "Muscle Spasticity," "Pain Management," "Treatment Outcomes," "Injection Therapy," "Adverse Effects," "Efficacy," "Safety," "Jaw Function," "Masticatory Muscle," "Clinical Trials," "Nocturnal Bruxism," "Sleep Bruxism."

The search string was crafted using a combination of these keywords and Boolean operators to refine the search effectively. The expanded search string is as follows: (("Botulinum Toxin Type A" OR "Botox") AND ("Temporomandibular Joint Disorders" OR "Temporomandibular Pain") AND ("Bruxism") AND ("Muscle Spasticity" OR "Pain Management" OR "Chronic Pain") AND ("Treatment Outcomes" OR "Adverse Effects" OR "Efficacy" OR "Safety" OR "Jaw Function" OR "Masticatory Muscle") AND ("Clinical Trials" OR "Therapeutic Use")) OR (("Botulinum Toxin, Type A" AND "Bruxism") AND ("Treatment Efficacy" OR "Functional Improvement" OR "Sleep Bruxism" OR "Nocturnal Bruxism" OR "Randomized Controlled Trials")).
